# Does omega-3 lower blood pressure? A protocol for systematic review and meta-analysis

**DOI:** 10.1097/MD.0000000000021955

**Published:** 2020-08-28

**Authors:** Li-Yu Tao, Yi-Ru Wang, Yi-Fan Zhang, Ping Liu, Xiao-Hong Chen

**Affiliations:** a Shuguang Hospital Affiliated to Shanghai University of Traditional Chinese Medicine; b Longhua Hospital Affiliated to Shanghai University of Traditional Chinese Medicine, Shanghai, China.

**Keywords:** blood pressure, hypertension, meta-analysis, omega-3, review

## Abstract

**Background:**

Hypertension is a clinically common cardiovascular disease, resulting in many complications. Omega-3 might be beneficial in lowering blood pressure. This protocol will be performed to evaluate the effects of omega-3 on blood pressure in hypertensive patients.

**Methods:**

We will search both the electronical databases and paper-published journals. Endnote software will be used to complete study screening and data extraction by 2 reviewers independently. Review Manager software will be used to synthesize the data. The primary outcomes are systolic blood pressure and diastolic blood pressure. Secondary outcome is the adverse effects.

**Results:**

The results of this study will propose a trustworthy evidence to evaluate the effects of omega-3 on blood pressure of hypertensive patients.

**Conclusion:**

The conclusion of our systematic review will reply whether omega-3 is an effectual intervention to lower blood pressure of hypertensive patients.

**Ethics:**

This review does not require ethical approval because all of the data analyzed in this review have been published.

**Registration number:**

INPLASY202070103 (DOI number: 10.37766/inplasy2020.7.0103)

## 1 Introduction

Hypertension has been regarded as a strong risk factor for stroke, coronary artery disease, and early mortality globally.^[1]^ The age-adjusted occurrence of hypertension among American adults ≥20 years’ old was approximated to be 46% from 2013 to 2016. Unfortunately, only 47% of those diagnosed with hypertension are satisfactorily treated.

Previous researches suggest that healthy diet and lifestyle, adequate physical exercise, reduced salt intake or omega-3, and folate supplementation can reduce blood pressure, enhance antihypertensive drug efficacy, and decrease cardiovascular diseases risk.^[2,3]^ The main ingredients of omega-3 could reduce systolic blood pressure,^[4,5]^ play anti-inflammatory and anti-thrombotic properties, and stabilize advanced atherosclerotic plaques.^[6,7]^ Moreover, there may be a dose–response effect of omega-3 on blood pressure.^[8]^ However, a prospective cohort study in 2019 suggested that l continuing dietary consumption of omega-3 fatty acid was not associated with happening hypertension. To our best known, the newest meta-analysis was published in 2016^[4]^ and there are some new studies after 2016 not included in the analysis.

Therefore, we intend to search global clinical research about omega-3 on blood pressure and systematically evaluate the effects of omega-3 supplement by meta-analysis method. The results will update the opinions of omega-3 on blood pressure and provide more scientific evidence for clinical strategy. In this meta-analysis protocol, we choose blood pressure as the major outcome.

## 2 Methods

We will follow the Cochrane Handbook for Systematic Reviews of Interventions and the Preferred Reporting Items for Systematic Reviews and Meta-Analysis Protocol (PRISMA-P) statement guideline^[9]^ and describe all the changes in the related article if needed.

### 2.1 Inclusion criteria

#### 2.1.1 Participants

All patients should meet the diagnostic criteria of hypertension established by the American College of Cardiology (ACC)/American Heart Association (AHA): all people with blood pressure >130/80 mmHg have hypertension.^[10]^ There will be no limitations of country, race, and comorbidity.

#### 2.1.2 Interventions

Patients in the experimental group should be given with omega-3 or omega-3 plus conventional treatment, such as thiazide or thiazide type diuretics, angiotensin converting enzyme inhibitors, angiotensin receptor blockers, beta blockers, and so on. It will not be limited to frequency, dose, and course of omega-3.

#### 2.1.3 Comparison

The patients in control group should be treated without omega-3.

#### 2.1.4 Outcomes

1. Primary outcomes: systolic blood pressure and diastolic blood pressure (mmHg).
2. Secondary outcome: adverse effects.

#### 2.1.5 Types of studies

All the randomized controlled clinical trials of omega-3 for hypertension will be included in this review.

### 2.2 Search methods for identifying the studies

#### 2.2.1 Data source

The search time limit is from the inception to September 2020. Two reviewers (LYT and YRW) will independently search all the potential studies according to the premade strategy by the following means: studies included in electronic databases (PubMed, Cochrane Library, Web of Science, China National Knowledge Infrastructure, Chinese Biological and Medical database and Wanfang Database); studies only published on paper (relevant journals, conference articles, and dissertations); unpublished researches to avoid missing gray literature.

The preestablished strategy is as follows.

1. Omega-3 [mh]
2. (n 3 Oils OR n 3 PUFA OR n-3 Oils OR Omega 3 Fatty Acids OR n-3 Fatty Acids OR Fatty Acid, n3 OR n 3 Fatty Acids OR n-3 Polyunsaturated Fatty Acid OR n-3 PUFA OR PUFA, n-3 OR PUFA, n3 OR n3 PUFA OR Omega-3 Fatty Acids OR n3 Oils OR n3 Fatty Acid OR n 3 Polyunsaturated Fatty Acid OR n3 Polyunsaturated Fatty Acid) [tw]
3. 1 OR 2
4. Eicosapentaenoic Acid [mh]
5. (omega 3 Eicosapentaenoic Acid OR omega-3-Eicosapentaenoic Acid OR 5,8,11,14,17-Eicosapentaenoic Acid OR 5,8,11,14,17-Icosapentaenoic Acid OR Eicosapentanoic Acid OR Acid, Eicosapentanoic OR Timnodonic Acid) [tw]
6. 4 OR 5
7. 3 OR 6
8. Hypertension [mh]
9. (Blood Pressures, High OR High Blood Pressures OR High Blood Pressure OR Blood Pressure, High) [tw]
10. 8 OR 9
11. 7 AND 10
12. randomized controlled trial [pt]
13. controlled clinical trial [pt]
14. randomized [tiab]
15. human trials as topic [mesh: noexp]
16. randomly [tiab]
17. trial [ti]
18. 12 OR 13 OR 14 OR 15 OR 16 OR 17
19. humans [mh] NOT animals [mh]
20. 18 and 19
21. 11 and 20

Pubmed search syntax

[mh] denotes a Medical Subject Heading (Mesh) term (“exploded")
[tw] denotes text word
[pt] denotes a Publication Type term
[tiab] denotes a word in the title OR Abstract
[sh] denotes a subheading
[mesh: noexp] denotes a Medical Subject Heading (Mesh) term (not “exploded")
[ti] denotes a word in the title.

#### 2.2.2 Search strategy

**Figure 1:**
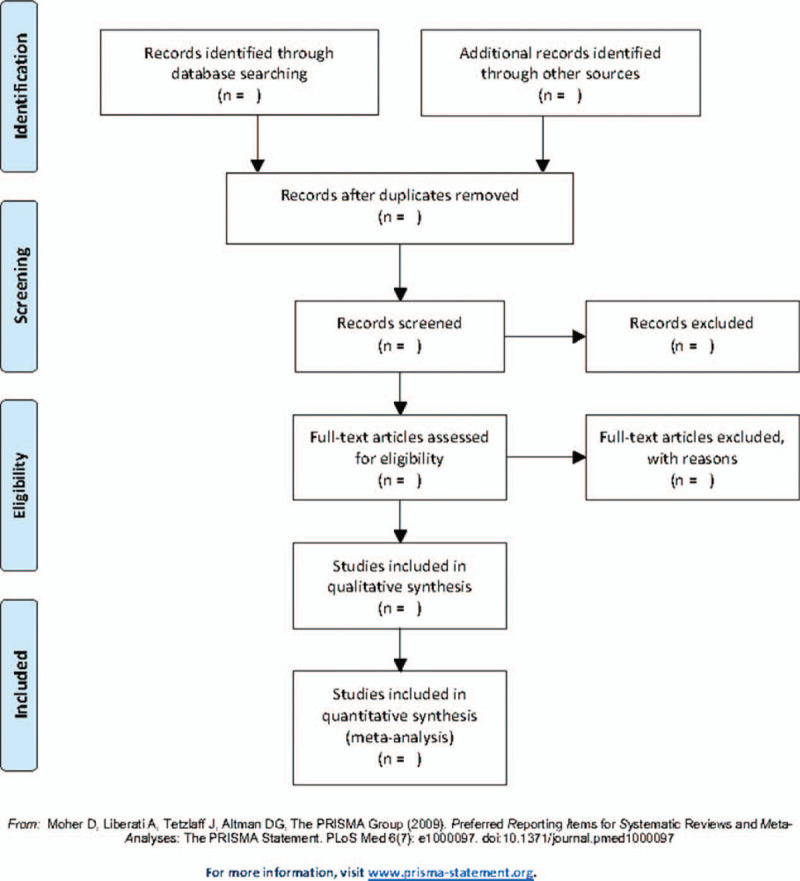
Flow diagram of study selection process.

We will export all the potential researches to the Endnote software (version 9.3.2, Thomas Reuters, CA) with duplicates being excluded by Endnote automatically. Two independent researchers (LYT and YFZ) will skim the titles and abstracts of the retrieved articles to complete the primary selection. Then the same 2 authors will read the full article to decide whether it could be included. A third author (PL) will check whether the selecting results are the same. If there is any disagreement, LYT and YFZ will discuss these and LP will make the final decision. The steps of study selection are listed in a flow diagram (Fig. 1).

### 2.3 Data extraction and synthesis

YRW and YFZ will use the Review Manager (Revman, version 5.3.5; The Nordic Cochrane Center, Copenhagen, Denmark: http://community.cochrane.org) software to extract literature data independently. A third party (XHC) will check whether the extracted data are accurate and consistent. We will contact the authors to request for a confirmation in the condition that data of studies are ambiguous, lost, or not extractable. The extracted data are as follows.

1. Basic information: title, first author name, published time
2. Patients: diagnostic criteria, ethnicity, age, duration of hypertension, and combined disease
3. Intervention: period, dose and frequency of omega-3, intervention of control group, combined drugs, and follow-up
4. Study designs: type, randomization, concealed and blinding method, sample size, baseline balance, completeness, and analysis of data
5. Primary outcome: systolic blood pressure and diastolic blood pressure
6. Secondary outcome: adverse effects

### 2.4 Risk of bias

LYT and YRW will evaluate the risk of bias by using Cochrane Collaboration's tool independently. There are 7 dimensions to assess which contain randomized sequence generation, concealed allocation, blinding of participants and personnel, blindness of outcome assessors, incompletion of outcome data, selection of reporting outcome, and other underlying bias. The Cochrane Handbook for Systematic Reviews of Interventions (Version 5.3) suggests 3 grades of each bias: low, unclear, and high level. We will search for a third author (XHC) when some disagreements are unable to be resolved. Or else, we will ask the Cochrane Professional Group for help and made the final decision.

### 2.5 Evaluation of heterogeneity

*χ*^2^ Test will be used to measure the homogeneity of included studies. If *I*^2^ value <50%, it represents that there is no or small statistical heterogeneity relatively; thus, a fixed-effect model will be used to complete the meta-analysis; If *I*^2^ value >50%, we will hold the view that a significant heterogeneity exits among the included studies, and a random-effect model will be used. To manage the significant heterogeneity, we will firstly examine the accuracy of all the raw data. Then the sensitivity will be analyzed to detect the origins of heterogeneity. Due to the following reasons, we will operate subgroup analysis to deal with the heterogeneity.

1. Different ages and races of patients.
2. Different duration of hypertension.
3. Different frequencies and courses of omega-3.
4. Tests with high risks of bias.

If it is still unable to diminish the heterogeneity, we will use descriptive analysis.

### 2.6 Reporting bias

If a meta-analyzed outcome contains >10 studies, funnel plots will be used to conclude the risk of reporting bias. No apparent publication bias was detected when the 2 sides of funnel plot are balanced. If it is unable to make a determinate decision according to the image, STATA software (version 12.0; Texas; https://www.stata.com/) will be adopted to make an Egger test for quantitative analysis.

### 2.7 Quality of evidence

We will use GRADE profiler software (Version 3.6, GRADEpro GDT, McMaster University, 2015; Evidence Prime, Inc; https://gradepro.org/) to measure the quality of evidence. There are 4 levels of the grade: high, medium, low, and extremely low.

### 2.8 Sensitivity analysis

We will examine whether low-quality studies included by performing the sensitivity analysis. This is a method to assess the reliability of the synthesized results and conclusion of meta-analysis. Each included article or some types of articles will be removed one by one then test the *I*^2^ value.

## 3 Discussion

The first meta-analysis about omega-3 treating hypertension indicated that diet supplementation with a relatively high dose of omega-3 could lead to clinically relevant blood pressure reductions. However, use of omega-3 as antihypertensive therapy will require demonstration of long-term efficacy and patient acceptability of lower doses.^[11]^ Another systematic review suggested that omega-3 is more effective in some cases than other lifestyle-related interventions (such as increasing physical exercise, limiting alcohol or reducing sodium intake) for lowering blood pressure among hypertensive populations without taking antihypertensive pill.^[5]^ However, it could not provide a convincing proof that there is a strong association of increased omega-3 consumption and reduced incidence of elevated blood pressure according to a review published in 2016.^[4]^ Some recent clinical trials proposed that omega-3 had a hypotensive efficacy in hypertensive patients.^[12,13]^ Therefore, this systematic review and meta-analysis might change the conclusion about omega-3 treating hypertension patients.

In this review, we will choose prospective study design to minimize the potential selection bias. However, there may be some possible limitations in this meta-analysis protocol. First, different doses, frequencies, and forms of omega-3 may result in significant heterogeneity and low methodological quality. Secondly, we will only include articles published in Chinese or English language, which may increase the selection bias. Thirdly, different regions and sexes of the hypertensive patients also could be a heterogeneity risk.

## Author contributions

XHC and PL contributed to the conception of the study. The manuscript protocol was drafted by LYT and was revised by YRW. The search strategy was developed by all the authors. LYT and YRW will independently search the data source and assess the risk of bias. LYT and YFZ will screen the potential studies and extract data from the include studies independently. YRW will complete the data synthesis and assessment of reporting bias, sensitivity analysis and the quality of evidence. XHC and PL will arbitrate in cases of disagreement and ensure the absence of errors. All authors approved the publication of the protocol.

**Conceptualization:** XHC and PL

**Formal analysis:** LYT

**Software:** YRW

**Supervision:** XHC and PL

**Writing – original draft:** LYT

**Writing – review & editing:** YRW
